# Stress during Commercial Hatchery Processing Induces Long-Time Negative Cognitive Judgement Bias in Chickens

**DOI:** 10.3390/ani11041083

**Published:** 2021-04-10

**Authors:** Louise Hedlund, Tiphaine Palazon, Per Jensen

**Affiliations:** Institution of Physics, Chemistry and Biology, Linköping University, 58183 Linköping, Sweden; louise.hedlund@liu.se (L.H.); tippa108@student.liu.se (T.P.)

**Keywords:** animal welfare, laying hens, hatchery, corticosterone, early stress, cognitive judgement bias

## Abstract

**Simple Summary:**

Worldwide, billions of laying hen chicks are incubated, hatched and processed in industrial hatcheries every year. When exposed to stress, hormones are incorporated in the feathers of the birds. Here, we measured levels of the stress hormone corticosterone to investigate possible stress during the incubation. Further, animals can perceive their environment either in a positive (optimistic) or a negative (pessimistic) way. We investigated how the early hatchery experiences affects “optimism” and “pessimism”. Commercially hatched chicks were exposed to a positive cue, an aversive cue, and ambiguous cues, in order to evaluate the cognitive welfare state of the animals. These chicks were compared to a group of non-stressed animals. Commercially incubated chicks did not have elevated levels of feather corticosterone, which implies that the main part of the stress effects from the hatchery originates from the period around hatch. Latencies to approach ambiguous cues were longer for the stressed chicks, i.e., these showed a more pessimistic-like behaviour. We conclude that the main part of the stress effects in commercially hatched chicks originates in the period around hatch, and further, that these birds show lasting levels of increased pessimism. This strongly indicates a long-time poorer welfare state for the animals.

**Abstract:**

Worldwide, billions of laying hen chicks are incubated and processed under highly industrialised circumstances every year, which, as we have previously shown, has long-lasting effects. Here, we measured corticosterone incorporated in down feathers to investigate possible stress during the incubation and showed that commercially incubated chicks did not have elevated levels of feather corticosterone, which implies that the main part of the stress effects from hatchery originates from the perinatal period and the handling immediately post-hatch. Further, we investigated how the early hatchery stress affects the chicks’ cognitive welfare state, i.e., “optimism” and “pessimism”. We exposed commercially hatched chickens to a positive cue, an aversive cue and ambiguous cues. The birds were tested at 1 and 10 w of age and the behaviour was compared with that of non-stressed chicks. Latencies to approach ambiguous cues were longer for the stressed chicks, both at 1 (*p* = 0.008) and at 10 (*p* = 0.020) weeks of age, i.e., these showed a more pessimistic-like behaviour. We conclude that the main part of the stress effects in commercially hatched chicks originates in the perinatal period, and further, that these birds show lasting levels of increased pessimism. This strongly indicates a long-time poorer welfare state for the animals.

## 1. Introduction

In commercial egg production, billions of chickens around the world are incubated and hatched under highly industrialised conditions that cause severe physiological stress during incubation as well as during the processing after hatching, and we have previously reported the behavioural effects of this even at 20 weeks of age [1,2]. Apart from behavioural effects, the stress experienced pre- and post-hatch also affects the chicks’ blood plasma corticosterone (CORT) levels and reactivity. In our previous study, chicks from a commercial hatchery had enhanced levels of circulatory CORT immediately after incubation when compared to control chicks, and this was further increased by the hatchery processing [1].

In commercial hatcheries, chickens are incubated in large, noisy incubators that can potentially induce stress already in the growing embryos. Plasma CORT is a common indicator of stress, but it can only be used to answer questions about the animals’ immediate response to an event. However, during incubation, the circulating CORT in the growing embryo is incorporated in the down feathers from when the chick starts to develop feathers at incubation day 7–8 [3,4]. Hence, measuring CORT in the down feathers during the first days after hatching will give an indication of the cumulative stress experienced during the incubation period.

Stress during the perinatal period has the potential to induce long-time modifications of cognitive states and welfare in both humans and other vertebrates [5]. For example, research on chickens shows that injections of CORT in the later part of the incubation period does affect memory in the hatchling [6,7]. Furthermore, Tahamtani and colleagues [8] showed that rearing of young chicks in a barren environment caused impaired working memory later in life. In contrast, stress-induced negative consequences can be reduced by increasing environmental complexity [9]. The question remains how the previously described hatchery stress affects the cognitive welfare state of chickens in the short as well as long perspective.

Humans are capable of explaining their emotional states verbally, however, this is obviously not possible for non-human animals. Instead, behaviour can be used as an indicator. One possible measure of this is the “cognitive judgement bias” (CJB), which refers to an individual’s propensity to show a positive or a negative response to an ambiguous stimulus [10]. This can also be defined as “optimism” and “pessimism” [11]. Typically, this is evaluated by training an animal to associate one cue with positive consequences and another cue with negative consequences, and thereafter observe the animal’s response to ambiguous cues with combined features of these. There have been several studies on how CJB in animals is affected by, and correlated to, stress. Calves show a more pessimistic behaviour in a CJB-test after dehorning and separation [12,13] and dogs scoring high on separation-related behaviours are more likely to make negative judgements [14,15]. Environmental enrichment has been shown to affect CJB, where, for example, pigs [16] and rats [17] housed in an enriched environment show more optimistic behaviours [16]. Studies of CJB in chickens (e.g., [9,18,19,20,21,22]) provide evidence that acute stress biases their judgment. For example, Seehuus and colleagues [19] showed that there are affective consequences of depriving chickens of foraging substrate, where deprived birds showed more negativity in the judgement of an ambiguous cue compared to controls. Furthermore, Oluwaseun and colleagues [20] observed that chickens fed with mealworms injected with CORT were more inclined to interpret an ambiguous stimulus as negative and Pichová [23] and colleagues showed that there is a relationship between feather pecking and CJB where chickens from a line bred on high feather pecking show a more pessimistic behaviour. Hence, we applied a CJB-test to investigate short- and long-term consequences of the perinatal stress associated with commercial hatchery routines.

A drawback with CJB-tests is that they usually require extensive training before testing, which makes them unsuitable for studying large groups of animals. This is often desired when evaluating, for example, different housing conditions and procedures in commercial practice. The CJB test we used here does not require any initial training before testing. Instead, it relies on the innate perception of social stimuli as attractive combined with a strong tendency to seek social contact with conspecifics, as well as the innate perception of a bird of prey as an aversive stimulus [21,22]. Hence, the approach tendency to either a mirror image, perceived as a live social partner, or an aversive owl can be compared to two-dimensional pictures of chicks and ambiguous morphed combinations of the pictures. In studies validating this test, chicks stressed by social isolation were more inclined to interpret the ambiguous cues as aversive compared to control animals [21,22]. This CJB study design, relying on the innate perception of a chick, was particularly suitable for this study since it was critical that the animals were handled as little as possible before testing, so as not to influence any potential stress effects from the handling in the hatchery. Furthermore, it allows for the testing of larger groups of animals.

To improve the understanding of commercial hatchery stress in chicks, we measured CORT levels in the down feathers collected after hatching, and performed CJB tests during the first week of life as well as 10 weeks later. We hypothesized that the birds from the commercial hatchery would have higher levels of CORT in their down feathers and furthermore, would be more “pessimistic” in their responses to the ambiguous stimuli than birds hatched and handled under calm conditions. We think that this study will provide insights into the industrially used chickens’ affective state and conception of the environment and the influence on their welfare.

## 2. Materials and Methods

### 2.1. Animals and Housing

All animals used in this study were White Leghorn chicks from the Lohmann LSL strain (Lohman Tierzucht, Germany). Experimental and control chicks were from the same parental flock, and from eggs collected during the same time period.

101 female chicks (hatchery chicks, HCs) were obtained from the commercial hatchery Gimranäs AB, Herrljunga, Sweden after processing according to standard routines, as previously described in Hedlund and colleagues [1]. Briefly, following hatching they were manually sex sorted and vaccinated, transported on conveyer belts, packed in boxes and transported for 3.5 h in a car. On arrival at the university, they were placed in rearing pens and, from this point on, treated the same way as the control chicks. The groups were kept in the same room, hence, were exposed to the same temperature, humidity, light cycle and potential unpredicted disturbances such as noise.

For control chicks, (CCs), 200 fertilized eggs from the same batch as HCs were collected before start of incubation and placed in a small incubator at Linköping University. After hatching, the chicks were removed from the incubator at the same time point as the HCs. They were handled carefully and quietly as they were sex sorted by wing inspection and the males were discarded. After sexing, the females (*n* = 88) were immediately placed in rearing pens. CCs were wing tagged and vaccinated for Marek’s disease at the age of three weeks. At the same time, HCs were wing tagged and sham vaccinated.

Throughout the whole experiment, birds from the two treatments were kept in separate but identical pens. From one to five weeks of age, they were evenly distributed in four identical 90 × 90 cm pens. At five weeks of age, chickens were moved to another facility and placed in two identical multilevel pens measuring 260 × 300 cm. The birds were kept on sawdust and provided with perches, nest boxes and ad lib food and water.

### 2.2. Down Feather Corticosterone

At four days of age, after the first behavioural tests, neck down feathers were collected from 38 randomly chosen chicks (HC, *n* = 20; CC, *n* = 18). The feathers were stored in plastic bags at room temperature until the time for analysis. For the analysis, each sample of −5 mg feathers were placed with a metal bead in a tube. The tubes were placed in liquid nitrogen for 2 min, whereafter they were placed in a Tissuelyser at 23 Hz for 2 min, in order to pulverize the samples. 1 mL of methanol was added to each sample and these were left overnight on a shaker at room temperature. The next morning, all samples were centrifuged, and 0.8 mL of the methanol extraction was transferred to new tubes. The new tubes were placed in a SpeedVac for the methanol to evaporate and each pellet was then dissolved in 250 µL assay buffer. The concentration of CORT was determined using an ELISA kit from ENZO Life Sciences (AH Diagnostics AB, Solna, Sweden) and tested in duplicates following the standard protocol. The intra-assay coefficient of variation was 3.1%.

### 2.3. Cognitive Judgement Bias

**Figure 1:**
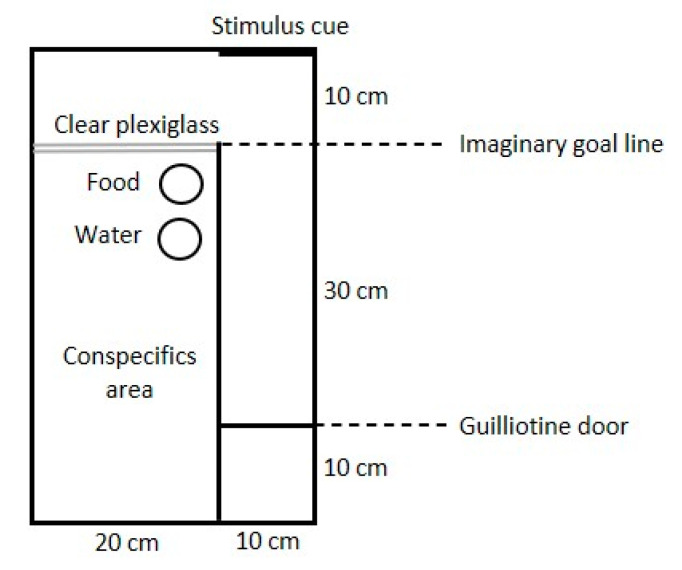
Arena used for 1-week-old chicks to measure cognitive judgement bias.

A cognitive judgement bias (CJB) assessment was conducted twice—during the first week and at 10 weeks of age—on 30 randomly chosen birds from each treatment (different birds at the two ages), in a total of 120 chickens. The arena used during the 1st week is shown in Figure 1 and the arena for the 10-week-old birds had the same design but was made ten times bigger. Briefly, the birds were released from a start area by lifting a sliding door and were then allowed to traverse a straight alley in order to gain contact with conspecifics. To do so, they had to pass a mirror or a picture at the end of the alley, as described below. Four chicks were placed in a companion compartment in one part of the arena, serving as social attraction, and one test chick was placed in a start box at the start of the alley. After 10 s, the start box was opened, and the test chick was free to move through the alley. All walls in the arena, except the clear plexiglass wall, were solid wood.

Each chick was tested on four different stimulus cues, with 1.5 h in-between the tests. In-between tests, the animals were returned to their home pen. First, all birds were tested with a mirror at the end of the alley, which is a strong attractive cue since a mirror image is perceived as if it is a live chicken [24]. The mirror test had two purposes in addition to serving as the most positive of the four stimuli; to allow the chicks to learn the procedure, and to ascertain that birds from both treatment groups had a similar motivation to move through the alley in order to join their conspecifics. Therefore, chicks that did not pass the goal line within 5 min were excluded and replaced with new chicks (*n* = 2).

Thereafter, all chicks were tested with three different pictures. Fifteen chicks from each treatment were first tested with a picture of a chick, secondly to a picture of an owl. The rest of the chicks were presented to the same cues, but in the opposite order. Lastly, all chicks were presented with an ambiguous cue—a morph between the chick picture and the owl picture. For each chick, we measured the time until they passed a goal line 10 cm in front of the stimulus. The maximum time for each test was 5 min and for chicks that did not reach goal line within this time, a latency of 5 min was scored.

**Figure 2:**
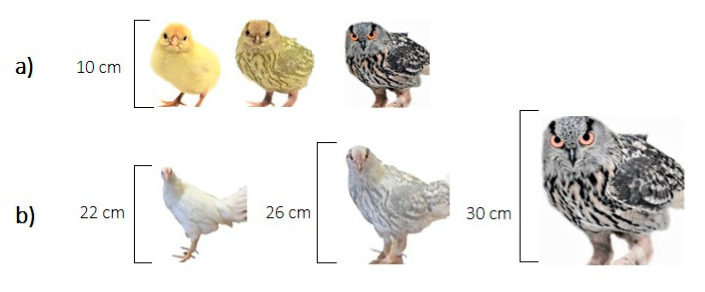
Stimuli used for measuring cognitive judgement bias at (**a**) 1, and (**b**) 10 weeks of age.

The pictures used as stimuli are shown in Figure 2. The most ambiguous stimuli were 50–50% morphs between the chicken and the owl pictures, created in the computer program Morpheus Photo Morpher v.3.17 Standard (Morpheus Development, Howell, MI, USA). The images were adjusted so that the chicken pictures were the same sizes as the tested chickens. All images were printed in colour with a white background and attached to a white wall at the end of the alley.

### 2.4. Statistics

For the CJB-test, an independent samples t-test was used to evaluate differences between latency to reach goal line for the mirror. To cater for individual variation in social motivation, latencies for the other cues were standardized against the latency in the mirror exposure (latencies were expressed as increased percent of time to reach the goal compared to the time to reach the mirror). To test for differences, these standardized values were tested in a paired samples *t*-test to evaluate differences between cues and in a generalized linear model, using normal distribution and link function “identity”, and with treatment and cue as factors. For the CORT data, an independent samples t-test was used to evaluate differences between the two treatments. All statistical analyses were run in SPSS.

## 3. Results

**Figure 3:**
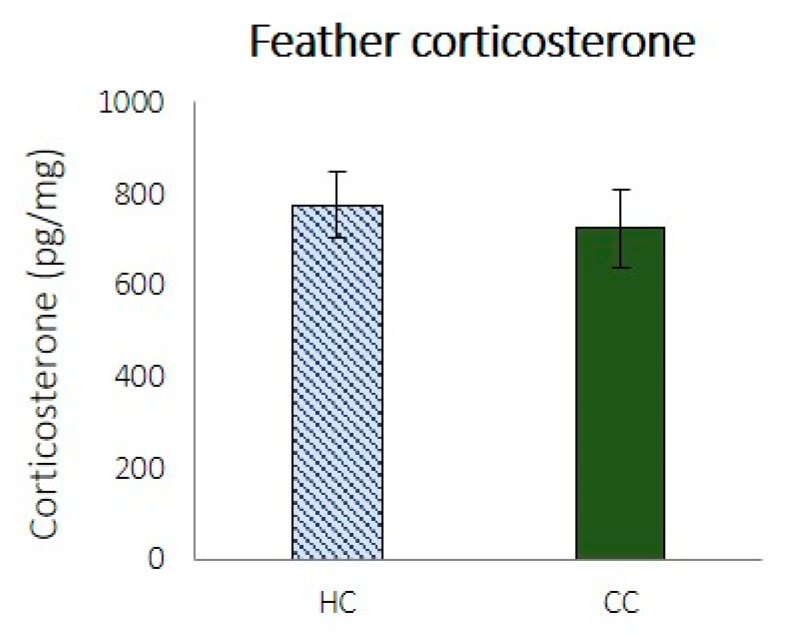
Down feather corticosterone in 4-day-old chicks, where HC—hatchery stressed chicks, and CC—control chicks.

The down feather CORT levels in HCs and CCs are shown in Figure 3. One severe outlier was removed from the HC group before testing and was not included in the averages shown in Figure 3. (CORT level: 2733 pg/mg feathers). There were no significant differences between the treatments (*t* (35) = 0.448, *p* = 0.657).

**Figure 4:**
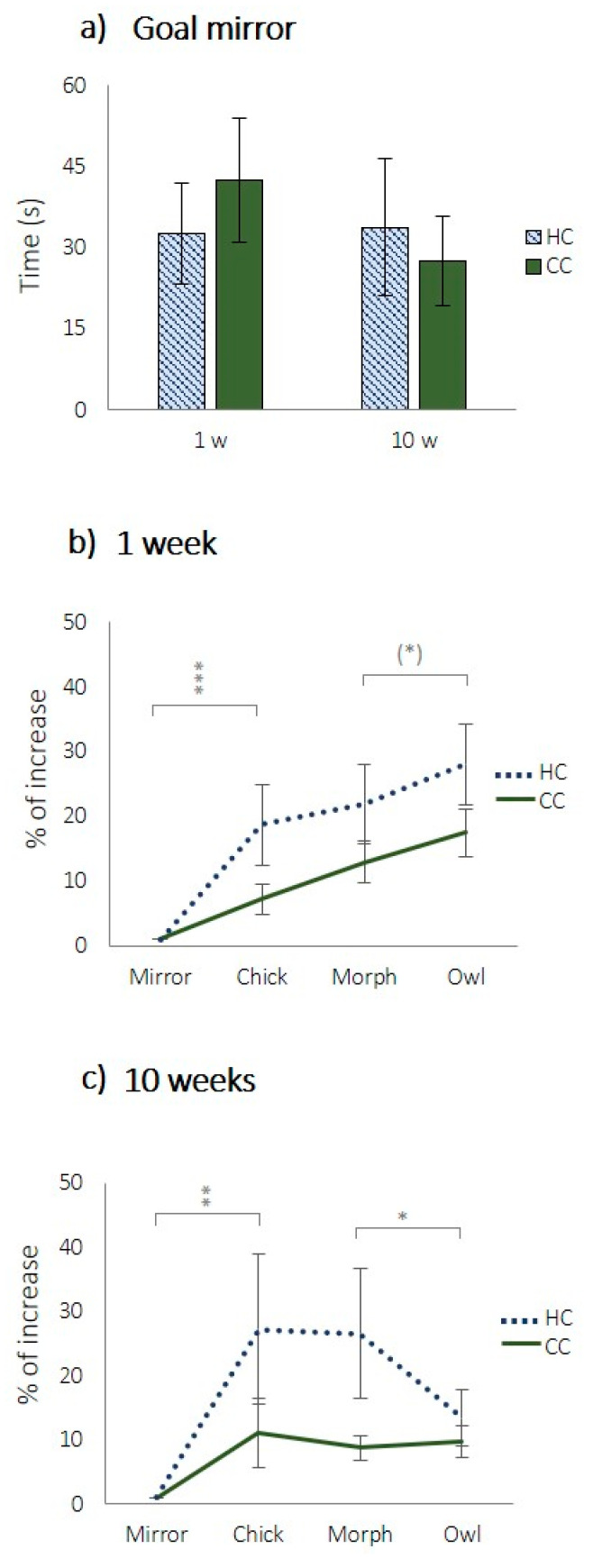
Latency to reach goal line in the straight alley maze in (**a**) seconds when exposed to mirror, and (**b**,**c**) in % increase compared to mirror at (**b**) 1, and (**c**) 10 weeks of age, where HC = hatchery stressed chicks, and CC = control chicks, (*) *p* < 0.1, * *p* < 0.05, ** *p* < 0.01, *** *p* < 0.001.

In the CJB-test, when tested with the mirror, there were no differences between treatments in latency to reach the goal line, neither at 1 week nor 10 weeks of age (Figure 4a, 1 week: *t* (58) = 0.671, *p* = 0.505; 10 weeks: *t* (58) = −0.024, *p* = 0.981). There were significant effects of the stimuli presented on the standardised time to reach the goal line (Figure 4b, χ^2^ = 29.847, *df* = 3, *p* < 0.001; Figure 4c, χ^2^ = 11.114, *df* = 3, *p* = 0.011). 

For 1 week of age, the differences between the mirror and the chick were statistically significant (Figure 4b, *t* (59) = −3.532, *p* < 0.001) but not the differences between the chick and the morph (Figure 4b, *t* (59) = −1.318, *p* = 0.193) or the morph and the owl (Figure 4b, *t* (59) = −1.754, *p* = 0,085). For 10 weeks of age, the differences between the mirror and the chick (Figure 4c, *t* (59) = −2.791), *p* < 0.01) as well as between the morph and the owl (Figure 4c, *t* (59) = 3.132, *p* < 0.05) were significant, however, not the difference between the chick and the morph (Figure 4c, *t* (59) = 0.396, *p* = 0.693). However, at both ages, there were significant overall treatment effects where HCs were overall slower to approach all ambiguous stimuli (the pictures) at both ages (Figure 4b, 1 week: χ^2^ = 6.973, *df* = 1, *p* = 0.008; Figure 4c, 10 weeks, χ^2^ = 5.448, *df* = 1, *p* = 0.020).

There were no significant effects of the interaction between the treatment and stimuli (Figure 4b, χ^2^ = 2.420, *df* = 3, *p* = 0.490; Figure 4c, χ^2^ = 3.531, *df* = 3, *p* = 0.317).

The complete dataset is available online at Table S1.

## 4. Discussion

Chicks exposed to the stressful conditions during commercial incubation, hatching and processing during their first hours of life showed a pessimistic cognitive judgement bias 4–5 days after hatch and this negative judgement bias was still present ten weeks after hatch. However, there was no difference between these chicks and control chicks in the levels of CORT incorporated in down feathers during incubation. This indicates that the noisy incubator conditions do not, in themselves, cause increased levels of chronic stress. Our results therefore suggest that it is the stress experienced around and shortly after hatching that causes persistent changes of the cognitive and emotional state of chickens, consistent with a state of poor welfare.

The down feathers in chicks start to develop at day 7–8 of incubation and the growth rate slowly and proportionally increases up to day 14–15 of incubation, when the highest growth rate occurs [4]. After this, the growth rate slowly and proportionally decreases [4]. Hence, the larger part of the chicks’ down feathers are developed around day 14–15 of incubation. The feathers in this study were cut as close to the skin as possible, however, since the down feathers on newly hatched chicks have a limited length, the main part of the feathers analysed must have developed around and before 15 days of incubation.

Circulating CORT can be measured in the embryo from at least 10 days of incubation [25], but the adrenocorticotropic hormone had been detected already at day 7 of embryonic development [26]. However, plasma CORT concentrations in the embryo increases distinctively at day 14–16 of incubation [25], i.e., during the same period as when most of the down feathers are developed.

We have previously shown that levels of plasma CORT in commercially hatched chicks are, compared to controls, elevated immediately after hatch, and this has lasting effects up to 20 weeks of age [1]. Furthermore, we found that a major part of the long-lasting stress effects are caused by events occurring before the processing at the hatchery [2]. Based on the results from the present study, we conclude that these stress effects mainly derive from the perinatal period and the time shortly after hatch. Hatchlings in a commercial hatchery are kept in the incubator up to 48 h post-hatch, where they are exposed to, for example, high noise levels and formalin fumigation before being removed from the incubator for further processing and transport. Hence, this environment in the hatcher probably is an important cause of long-term stress effects on the chicks.

CJB is a common method to assess emotional states of animals [10]. It usually requires that the test subjects are trained to associate one stimulus with a positive outcome (usually food) and another stimulus with a negative outcome (e.g., no food). Once trained, their reactions to an ambiguous stimulus, combining features of the positive and the negative ones, are recorded. The training normally takes a long time, which makes the method unsuitable for studying many animals at a specific age. Hence, we used the method developed by Salmeto and colleagues [21], which relies on the innate propensities of chickens to maintain social contact, and to instinctively avoid predators. It can therefore be applied without previous training. The method has been validated and found to reliably induce a pessimistic response following acute stress [21].

Mirror images have a calming and attractive effect on chickens, probably because they are perceived as conspecifics [24]. We therefore used a mirror as the most positive stimulus and found no difference between HCs and CCs in latency to approach it during the testing. This means that neither of the groups were more fearful of the test situation or had impaired abilities to move through the alley of the arena, and that both groups had similar motivation to seek out conspecifics. The owl was the most negative of the stimuli, and both groups took longer to approach it at both 4–5 days and 10 weeks of age compared to the mirror. The chick image constituted a near-positive and the morph a near-negative ambiguous stimulus, and the HCs were slower to approach both, demonstrating a clear negative judgement bias.

When tested at 10 weeks of age, HCs approached the owl cue faster than both the chick and the intermediate cue, hence, the latencies of HCs at week 10 do not follow the same pattern as during week 1. This result was not in accordance with our hypothesis but could be explained by the fact that we cannot be sure about how the chicks perceive the pictures. It is possible that they do not perceive the owl cue as a threat at this age. Another explanation would be that they do, but perceive the morphed picture as even more threatful. However, even if there was no difference between treatments in the latency to approach the owl picture, there was still an overall effect of stimuli, showing that the pictures were all in a sense perceived as ambiguous. The fact that HCs were slower to approach ambiguous stimuli compared to CCs indicates an overall more pessimistic response.

Previously, Deakin and colleagues [18] showed a negative judgement bias in hens exposed to temperature stress and Oluwaseun and colleagues [20] found that broiler chickens were more pessimistic after the administration of CORT. Comparable results have been found in starlings which showed a positive judgement bias when kept in enriched cages [11] and negative judgement bias after having experienced a decline in environmental enrichment [27]. However, Pichová and colleagues [23] found no correlation between CJB and CORT levels, implying that the relationship between high CORT levels and pessimism might not be as simple. However, to our knowledge, this is the first study that shows a long-term cognitive effect of the typical processing procedures in a commercial hatchery, which are considered to be acutely stressful [1].

CJB as a valid measurement of animal emotions and welfare has been extensively studied and reviewed. Mendl and colleagues [10] conclude that cognitive bias, and in particular judgement bias tasks, can be used when assessing in animals. Animals in negative affective state will be more likely to respond with fear or hesitation to ambiguous cues. This mirrors a pessimistic conception of the environment and will affect the animals’ emotions and welfare.

Billions of chickens are hatched under industrial and stressful conditions every year and may therefore perceive a long-term state of poor welfare. This is consistent with our earlier studies, showing that commercially hatched chickens have long-term elevated physiological stress responses and possibly show more feather-pecking as adults than chicks hatched under calm conditions [1].

## 5. Conclusions

We conclude that the highly industrialised conditions during commercial hatching result in more pessimistic birds and that this effect lasts for at least 10 w, indicating a long-lasting reduced cognitive welfare state. Furthermore, we conclude that a major part of the stress effects from commercial hatchery chicks originates from experiences during the perinatal period rather than earlier during incubation.

## Data Availability

The data presented in this study are available in Supplementary Table S1.

## Supplementary Materials

The following are available online at https://www.mdpi.com/article/10.3390/ani11041083/s1, Table S1: Complete data set.
